# Impact of training in the use of an early warning system on in-hospital cardiac arrests

**DOI:** 10.1186/cc9897

**Published:** 2011-03-11

**Authors:** A Raj, J Zwaal

**Affiliations:** 1 Kingston Hospital NHS Trust, Kingston upon Thames, UK

## Introduction

The introduction of an early warning system (EWS) has been associated with a reduction in in-hospital cardiac arrest (CA) [1]. We set out to determine the impact of a programme of training in the use of an EWS on the number and nature of CAs in our hospital.

## Methods

We conducted a retrospective chart survey of all adult CA patients pre and post implementation of a training programme in the use of the EWS. If a patient develops abnormalities in two or more physiological parameters, the system forces escalation of care through three levels of care, with involvement of junior medical staff at level 1 and senior ICU medical staff at level 3. Abnormal physiology was defined as: SaO_2 _< 90%, HR <50 or ≥110/minute, systolic blood pressure <90 mmHg, conscious level: only responsive to pain, respiratory rate <10 or ≥25/minute or clinical concern about the patient. Outcome parameters: CA/1,000 bed-days, percentage of CPR attempts deemed inappropriate by two senior intensivists, percentage of patients (in whom CPR attempts were deemed appropriate) with abnormal physiology prior to CA and survival post CA. Charlson's co-morbidity index (CCI) [2] was calculated for both periods. Differences between mean values were tested with Student's *t*-test and differences between percentages were tested according to the method described by Armitage [3].

## Results

After adjusting for age (mean pre: 81.7, post: 81.8 years (*P *= 0.99)), sex and co-morbidity (CCI pre: 6.4, post: 6.66 (*P *= 0.79)): CA/1,000 bed-days pre: 0.89, post: 0.76 (*P *= 0.24); percentage of inappropriate CPR attempts pre: 62.5%, post: 33% (*P *= 0.11); percentage of cases with abnormal physiology identified prior to arrest pre: 68.8%, post: 75% (*P *= 0.72); and survival pre: 12.5%, post: 0% (*P *= 0.20).

## Conclusions

Training in EWS was associated with a reduction in the number of CAs and percentage of inappropriate CPR attempts, both of which are in keeping with the literature. However, there was no significant difference in the percentage of cases with abnormal physiology identified prior to CA between both periods and there was no survival benefit after CA. An early warning tool may be unable to prevent CA in a subset of patients with deranged physiology.
